# Identification and evaluation of resistance to powdery mildew and yellow rust in a wheat mapping population

**DOI:** 10.1371/journal.pone.0177905

**Published:** 2017-05-23

**Authors:** Lijun Yang, Xuejiang Zhang, Xu Zhang, Jirui Wang, Mingcheng Luo, Mujun Yang, Hua Wang, Libo Xiang, Fansong Zeng, Dazhao Yu, Daolin Fu, Garry M. Rosewarne

**Affiliations:** 1 College of Life Sciences, Wuhan University, Wuhan, China; 2 Institute for Plant Protection and Soil Science, Hubei Academy of Agricultural Sciences (HAAS), Key laboratory of Integrated Pest Management on Crop in Central China, Ministry of Agriculture, Wuhan, China; 3 Institute of Biotechnology, Jiangsu Academy of Agricultural Sciences (JAAS), Nanjing, China; 4 Triticeae Research Institute, Sichuan Agricultural University, Wenjiang, Chengdu, Sichuan, China; 5 Department of Plant Sciences, University of California Davis, Davis, CA, United States of America; 6 Food Crops Research Institute, Yunnan Academy of Agricultural Sciences (YAAS), Kunming, China; 7 State Key Laboratory of Crop Biology, Shandong, Key Laboratory of Crop Biology, Shandong Agricultural University, Tai’an, China; 8 International Maize and Wheat Improvement Centre (CIMMYT) c/o Crop Research Institute, Sichuan Academy of Agricultural Science, Jinjiang, Chengdu, China; Western Australia Department of Agriculture and Food, AUSTRALIA

## Abstract

Deployment of cultivars with genetic resistance is an effective approach to control the diseases of powdery mildew (PM) and yellow rust (YR). Chinese wheat cultivar XK0106 exhibits high levels of resistance to both diseases, while cultivar E07901 has partial, adult plant resistance (APR). The aim of this study was to map resistance loci derived from the two cultivars and analyze their effects against PM and YR in a range of environments. A doubled haploid population (388 lines) was used to develop a framework map consisting of 117 SSR markers, while a much higher density map using the 90K Illumina iSelect SNP array was produced with a subset of 80 randomly selected lines. Seedling resistance was characterized against a range of PM and YR isolates, while field scores in multiple environments were used to characterize APR. Composite interval mapping (CIM) of seedling PM scores identified two QTLs (*QPm*.*haas-6A* and *QPm*.*haas-2A*), the former being located at the *Pm21* locus. These QTLs were also significant in field scores, as were *Qpm*.*haas-3A* and *QPm*.*haas-5A*. *QYr*.*haas-1B-1* and *QYr*.*haas-2A* were identified in field scores of YR and were located at the *Yr24/26* and *Yr17* chromosomal regions respectively. A second 1B QTL, *QYr*.*haas-1B-2* was also identified. *QPm*.*haas-2A* and *QYr*.*haas-1B-2* are likely to be new QTLs that have not been previously identified. Effects of the QTLs were further investigated in multiple environments through the testing of selected lines predicted to contain various QTL combinations. Significant additive interactions between the PM QTLs highlighted the ability to pyramid these loci to provide higher level of resistance. Interactions between the YR QTLs gave insights into the pathogen populations in the different locations as well as showing genetic interactions between these loci.

## Introduction

Powdery mildew (PM) and yellow rust (YR), caused by *Blumeria graminis* f. sp. *tritici* (*Bgt*) and *Puccinia striiformis* f. sp. *tritici* (*Pst*) respectively, are the most devastating diseases of wheat (*Triticum aestivum* L.) in cool climate regions [1,2]. Approximately 6 million ha of wheat in China is grown in areas prone to PM or YR epidemics. Major epidemics occurred in these regions in 1990 with grain yield losses due to PM epidemics estimated at 1.4 million tonnes [3] and to YR at 2.65 million tonnes [2,4]. Growing resistant cultivars is an effective, economical and environmentally safe approach to control these diseases [5].

Plant disease resistance can be classified as either qualitative or quantitative [6]. Qualitative resistance is generally conferred by single genes with large effects against the pathogen and can be observed in both seedling and adult plant stages. To date, 50 loci containing 78 resistant genes/alleles to PM [7,8] and 74 genes (*Yr1*–*Yr74*) to YR [9,10] have been identified in bread wheat and its relatives. This type of resistance has a strong tendency to be overcome by new races, particularly when a single gene is deployed over large areas. However while the gene remains effective, it strongly affects the presence and frequency of specific pathotypes in the field [11–13]. The majority of this type of resistance loci have been overcome by the pathogen with only a few, including *Pm2*, *Pm4*, *Pm21* and *Pm30*, still effective against prevailing *Bgt* isolates [13,14]. In YR, only *Yr5*, *Yr10* and *Yr15* are still effective against the prevalent Chinese *Pst* races of *Pst-CRY32*, *Pst-CRY33* and *Pst-V26* [15].

In contrast, quantitative resistance is mediated by multiple genes or quantitative trait loci (QTLs) [6] and is most commonly observed in adult plants grown under field epidemic conditions. This type of locus often show partial but additive effect against the majority of isolates, and is considered to be broad-spectrum and durable [6], making it highly valuable to breeding programs. Although individual adult plant resistance (APR) genes or QTLs often confer partial and inadequate resistance, combinations of such genes can result in “near-immunity” [16,17]. So far, 119 resistance QTLs for PM [18] and more than 140 QTLs for YR [19] have been identified, with nearly every chromosome harboring at least one resistance locus.

Wheat cultivars containing combinations of effective resistance genes are likely to provide long-lasting control of PM and YR diseases. Cultivar XK0106 showed high level resistance to PM [20] and YR [21] in both seedling and adult stages, while E07901 exhibited APR to both PM and YR [21]. The two cultivars are promising breeding sources with favorable agronomic traits, but little is known about the genetic basis of their resistance to both diseases. The objectives of this study were to 1) map QTLs responsible for resistance to PM and YR in a population derived from E07901 and XK0106 with SSR and SNP marker genetic linkage maps and 2) assess effectiveness of the detected QTLs alone or in combination in different environments.

## Materials and methods

### Plant materials and pathogen isolates

A total of 388 DH lines were developed from a cross between wheat cultivar XK0106 and E07901. The population was generated through the maize pollination technique [22] in Yunnan Academy of Agricultural Sciences, Kunming, China. A subpopulation of 91 DH lines were randomly selected from the 388 lines. Further sub-populations of 24 lines for PM and 21 for YR were selected based on their QTL complements and used for the evaluation of QTL efficacy against PM and YR separately in four different environments. Chancellor and Mingxian 169 were used as susceptible checks in PM and YR field trials respectively. Thirty lines with known *Pm* (S1 Table) and four near-isogenic lines containing key *Yr* genes (S2 Table) were used in seedling tests.

Isolate *Bgt*6-11, being incompatible with XK0106 and compatible with E07901, was used for the PM seedling assays, while five Chinese *Pst* races including *Pst-CYR29*, *Pst-CYR32*, *Pst-CYR33*, *Pst-Su11-4* and *Pst-V26* were used for the YR seedling assays. The first four of these races have been predominant in China since the 1980s [4], and the race *Pst-V26* was first isolated from Chuanmai42 in 2008 and is virulent to *Yr24/26* [23]. Sixteen differential *Bgt* isolates were listed in S1 Table.

### Seedling assays for PM and YR

Seedling resistance assays for PM were evaluated using a detached leaf segment method. Seeds of XK0106, E07901, F_1_, Chancellor and the DH lines were germinated and planted in square pots of 12×12×12 cm and grown to the two-leaf stage (10 days after planting). Leaf segments, 3 cm in length, were cut from the middle part of the primary leaf and placed on 0.5% water agar (w/v) supplemented with 50 mg L^-1^ Benzimidazole in clear plastic boxes with the abaxial epidermis facing upwards. Three independent replicates were used for each DH line. Inoculation was performed by blowing the spores into a plastic tower at a density of 4×10^3^ conidia cm^-2^. The leaf segments were then incubated in a growth cabinet with 80% relative humidity and a 12 h light 12 h dark photoperiod at 18±1°C. Infection type (IT) was scored on a 0–4 scale [24] at 12 days post inoculation (dpi), when the susceptible control Chancellor showed fully developed disease symptoms (IT 4). All lines were classed into two groups according to IT with resistant lines scoring between zero and two, and susceptible lines scoring three or four. XK0106, E07901 and a set of lines with known *Pm* gene to 16 differential *Bgt* isolates were also evaluated using the above method (S1 Table).

Seedling resistance assays for YR were evaluated under controlled greenhouse conditions. Thirty seeds of parents, Mingxian169 and single gene lines were sown separately in square pots of 12×12×12 cm. Seedlings at the two-leaf stage (14 days after planting) were inoculated with urediniospores of the five respective *Pst* races. The inoculated plants were incubated at 10 ± 1°C in a dew chamber in the dark for 24 h, and then transferred to a greenhouse at 17 ± 2°C. IT was scored 20 days after inoculation using a 0–9 scale described by Line and Qayoum [25]. Plants with an IT of 0 to 3 were considered resistant, 4 to 6 considered intermediate and 7 to 9 susceptible.

### Adult-plant assessment of PM and YR

Field trials for the QTL analysis were conducted at the Hubei Academy of Agricultural Sciences Nanhu farm (30.2856 N, 114.1839 E, 27 m) in Wuhan, Hubei Province. The PM trials were conducted during the wheat cropping seasons of 2011, 2012 and 2013 while the YR trials were conducted in 2010 and 2013. Trials had two replicates and were designed as randomized complete blocks with repeating checks. Each plot consisted of two 1.5 m rows with row spacing of 25 cm. Approximately 100 seeds were sown in each row and the susceptible check, Chancellor or Mingxian169, was planted every 20 plots and around the test lines to ensure ample PM or YR inoculum. The spreader rows of the susceptible checks were exposed to artificial inoculation of PM at stem elongation (Growth stage 30 according to [26]) with mixed conidia from the 16 *Bgt* isolates that were used in the seedling tests. A similar approach was adopted for YR with mixtures of *Pst-CYR32* and *Pst-CRY33* urediniospores suspended in the light weight mineral oil Soltrol 170 (Chempoint.com) applied to the spreader plots at the tillering stage (Growth stage 25, [26]). Powdery mildew severity (PMS) or yellow rust severity (YRS) was scored at the mid-grain filling stage (Growth stage 75, [26]). The three upper leaves of 15 randomly selected plants were assessed using a 0–9 scale [27] for PMS and a modified Cobb scale [28] for YRS. Disease severity of 15 plants was averaged to obtain the mean PMS or YRS for each plot.

Further field evaluations were conducted on selected lines from the DH populations that contained different combinations of QTLs. PM evaluations were conducted during 2014 at four sites including Nanhu farm in Wuhan, Wolong farm (32.0157 N, 111.5950 E, 69 m) in Xiangyang, Jiangbei farm (30.1407 N, 112.1953 E, 34 m) in Jingzhou and Meijiadun farm (30.4216 N, 115.0434 E, 49 m) in Huanggang, all located in Hubei Province. The YR evaluations were carried out during 2015 at four sites of Nanhu farm, Wuhan and Wolong farm, Xiangyang in Hubei Province, Taoyuan farm (25.0819 N, 102.4520 E, 1903 m), Kunming in Yunnan Province and Gangu farm (34.4508 N, 105.1842 E, 1278 m), Gangu in Gansu Province. Each DH line was planted in a 6.67m^2^ plot in randomized complete block designs containing three replicates. Detailed assessment was conducted at five sampling points in each plot with the severity of the upper three leaves of 20 plants assessed at each sampling point.

All locations were on field stations owned by the research organizations and external permission was not required to conduct the experiments.

### Statistical analysis

Chi-square analysis was performed to predict the minimum number of loci contributing to resistance against PM in XK0106 according to the segregation ratio of IT to isolate *Bgt6-11*.

### Genomic DNA extraction, SSR and SNP genotyping

Young leaves of the parents and DH lines were collected and frozen in liquid nitrogen. Genomic DNA was extracted using the CTAB protocol [29]. Three hundred and ninety-five SSR markers were randomly selected from the 21 Somers consensus chromosome maps [30] to test for polymorphisms between the parents. All of the polymorphic SSR markers were used to analyze genotypes of the 388 DH lines. Two STS markers, C*INAU15* [31] and *CINAU17* [32], and one EST-SSR marker *Xedm129* [33] associated with *Pm21* were also evaluated in the population.

The PCR assays for the SSR, EST-SSR and STS markers were conducted in an EDC-810 PCR Thermocycler (Dongsheng, Beijing, China) in a reaction mixture (10 μL) containing 10 mM Tris–HCl, pH 8.3, 50 mM KCl, 1.5 mM MgCl_2_, 0.2 mM dNTPs, 25 ng of each primer, 50 ng genomic DNA and 0.75U Taq DNA polymerase. Amplifications were performed at 94°C for 5 min, followed by 40 cycles at 94°C for 45 s, 50–60°C (depending on specific primers) for 45 s, and 72°C for 1 min, with a final extension at 72°C for 10 min. The PCR products (2 μL) were mixed with an equal amount of loading buffer and separated on 8% non-denaturing polyacrylamide gels (39 acrylamide: 1 bisacrylamide). Gels were silver stained and photographed.

The genotyping of the sub-population was conducted at the Genome Center of the University of California, Davis. The DNA of 80 DH lines and the parents were extracted and then genotyped through the 90K Illumina iSelect SNP array [34] following the manufacturer’s protocol. SNP allele clustering and genotype calling was performed with Genome Studio software v2010.3. Each of the SNP clusters were manually examined to correct imperfect calling of automated clustering. SNP markers with ambiguous SNP calling between parents and/or with a negative hybridization response in most lines were removed from the data set.

### Genetic map construction and QTL analysis

Initial linkage group (LG) construction using the polymorphic SSR markers was completed with Joinmap 4.0 [35]. Linkage analysis and marker ordering were carried out using the regression mapping algorithm with a threshold log-likelihood (LOD) ratio ≥3.0 with the recombination values being converted to genetic distances using the Kosambi mapping function. LGs were assigned to chromosomes by reference to Somers consensus maps [30].

Initially, mean PMS and YRS of all the lines from each year were used to identify QTLs with the SSR LG map. Composite Interval Mapping (CIM) was performed with WinQTL Cartographer version 2.5 [36] using Model 6 with five markers as controls and employing a window size of 10 cM. Significant thresholds for QTL detection were calculated for each dataset using 1,000 permutations with a genome-wide error rate of 0.05. Phenotypic variance (R^2^) explained by a QTL was obtained by the square of the partial correlation coefficient. Genetic maps were drawn using MapChart2.2 (http://www.wageningenur.nl/en/show/Mapchart.htm).

The SNP marker-LGs were constructed using MultiPoint software (http://www.multiqtl.com). Prior to map construction, all non-polymorphic SNP markers between parents as well as those markers with greater than 20% missing data were omitted. Eleven lines with poor quality data were also omitted. Segregation of the remaining SNP markers were subjected to Chi-square tests and severely distorted markers deviating from the expected segregation ratio (1:1) at the probability level *p* = 0.001 were excluded from further analyses. A maximum threshold rfs value of 0.05 to 0.15 with a 0.01 step was used to initially group the markers into different LGs. Multipoint linkage analysis of loci within each LG was then performed with the maximum likelihood (ML) mapping algorithm and the marker order was further verified through re-sampling for quality control via jack-knifing [37]. Markers with known chromosomal locations on the 90K_consensus_map ([34]; http://wheat.pw.usda.gov/cgi-bin/graingenes/report.cgi?class=mapdata;name=Wheat_2014_90KSNP) were used to assign LGs to chromosomes. Redundant SNP linked markers were removed with the remaining SNP markers being outlined in S3 Table. The complete marker dataset is supplied in S4 Table. These were also used to draw chromosome maps using MapChart 2.2 (http://www.wageningenur.nl/en/show/Mapchart.htm). This approach was repeated with the combined sets of SSR and SNP markers.

QTLs were mapped on the combined marker LGs using the phenotypic data of 80 remained DH lines of PM and YR. CIM was performed with WinQTL Cartographer version 2.5 [36] with the same parameters as described above. Significant thresholds for QTL detection were calculated for each dataset using 1,000 permutations with a genome-wide error rate of 0.05 (significant) and 0.1 (suggestive). Phenotypic variance (R^2^) explained by a QTL was obtained by the square of the partial correlation coefficient.

## Results

### Phenotypic evaluations of PM and YR in seedling tests

XK0106 and F_1_ lines of the cross between E07901 and XK0106 were highly resistant to *Bgt*6-11 (IT 0), whereas cultivar E07901 was highly susceptible (IT 4). The seedling assay of the DH population segregated in a 1:1 ratio (Table 1), indicating that a single gene was involved in XK0106 resistance. This was supported by the IT data for this population as it fitted a U-shaped frequency distribution. However there were a number lines with ITs of 1 to 3, suggesting the possibility that other minor QTLs may have been present in altering seedling IT.

**Table 1:** Genetic analysis of seedling resistance to *Bgt6-11* in a doubled haploid (DH) population derived from E07901 × XK0106.

	No. of plants/lines	Infection type						R: S ratio	Expected ratio	χ^2^
		0	0;	1	2	3	4			
**XK0106**	20	20								
**E07901**	20						20			
**F1**	20	20								
**DH**	388	176	0	15	15	47	135	1: 0.88	1: 1	1.36**

** significant at *P* = 0.01.

Values for significant at *P* = 0.01 is 6.63 (1:1).

The PM reaction patterns of XK0106 and E07901 to 16 differential *Bgt* isolates were compared with those of lines possessing known genes. The responses of XK0106 showed an identical pattern to that of Yangmai5/sub.6v (*Pm21*) with immunity (IT 0) to all the test isolates, while E07901 was susceptible (IT3 or 4) to all isolates except *Bgt*E01 (S1 Table).

The YR seedling ITs showed that XK0106 was resistant (IT 0–3) to four out of five *Pst* races but susceptible to *Pst-V26* (IT 8). This compatible reaction was similar to that observed in the Avocet *Yr26* NIL. E07901 was susceptible or moderately susceptible to all of the races tested, indicating that it doesn’t have *Yr10*, *Yr15* or *Yr24/26* (S2 Table).

### Phenotypic evaluation of PM and YR at the adult-plant stage

**Fig 1:**
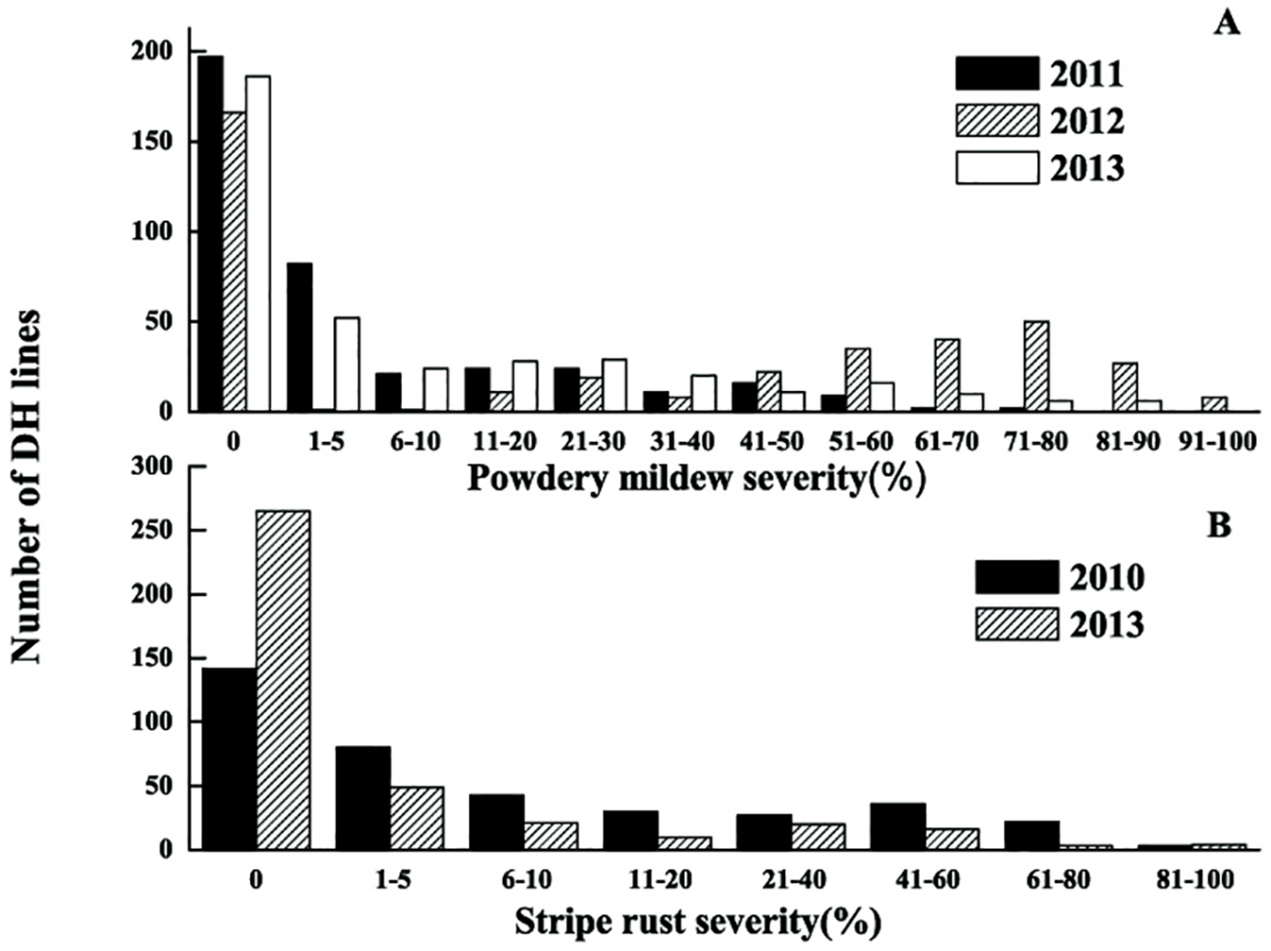
Frequency distribution of disease severity. Number of individuals from the E07901 × XK0106 doubled haploid population occurring in classes of disease severity for (A) powdery mildew severity and (B) yellow rust severity.

The mean severity of PM varied from 7.9% to 34.7% over the three years of testing, with 2012 being the highest (Table 2). XK0106 maintained its immunity in all of these seasons while E07901 ranged between 11.8% and 61.5%. The PMS of the 388 DH lines showed an L-type distribution with approximately half of the lines having a severity of zero while the rest of the lines were continuously distributed (Fig 1A). A number of lines consistently had a higher PMS than that of the susceptible parent E07901 (Table 2).

**Table 2:** Summary of disease severity scores of 388 DH lines and their parents grown in Wuhan in the years indicated. Disease scores are the leaf area covered by powdery mildew or yellow rust from two replicates.

				Population								
Year	XK0106		E07901	Minimum	Maximum	Mean	Std. Error of Mean			Skewness		Kurtosis
**Powdery mildew**												
**2011**	0		11.8	0	72.7	7.9		0.8	2.1		3.9	
**2012**	0		61.5	0	96.5	34.7		1.7	0.2		-1.6	
**2013**	0		33.4	0	87.3	13.4		1.1	1.7		2.2	
**Yellow rust**												
**2010**	0		10.0	0	100	17.0		1.3	1.6		1.2	
**2013**	0		26.5	0	100	6.0		0.8	3.3		11.8	

XK0106 was immune in both of the environments tested while E07901 had YRS scores of 10.0 and 26.5%. Mean YRS of the 388 lines in the DH population ranged between 6.0 and 17.0% and the severity of single DH lines varied between 0 to 100% in each of the two years (Table 2). The frequency distribution of DHs for YRS was continuous with a pronounced skewness towards resistance (Fig 1B).

### Genetic linkage mapping and QTL analysis

Overall, 134 (33.9%) of the 395 SSR markers showed polymorphisms between the parents. This was further reduced to 117 useful markers when run on the entire population and led to the construction of a genetic map with 26 linkage groups. Apart from chromosomes 1A and 1B, all other wheat chromosomes had between one and three LGs. Chromosome 3B had the greatest coverage with ten SSR markers, while chromosomes 3A, 4B and 6D had the least with only 2 markers each. Chromosome 2B had the longest genetic distance (130.5 cM), while chromosome 5A had the shortest distance (3.9 cM) (S3 Table).

The SNP marker set dramatically increased the marker density and chromosome coverage. In all, 11,746 (14.4%) out of 81,587 SNPs showed polymorphisms between parents and 11,330 could be incorporated into the map (including the 117 SSR markers). This resulted in 33 LGs and each was assigned to the different chromosomes of wheat according to 90K_consensus_ map information [34]. Each chromosome contained at least 1 LG and the group D chromosomes had the least representation of markers (225). The genetic map spanned 3,351 cM with an average density of one marker every 2.5 cM. Chromosomes 5B and 4D had the largest (119) and the fewest (14) number of markers, respectively. Chromosome 5A had the longest genetic distance (266.9 cM) and chromosome 4D had the shortest genetic distance (48.7 cM) (S3 Table).

Composite interval mapping was conducted on both the SSR map containing 117 markers from 388 lines as well as the SSR+SNP map containing 11,330 marker from 80 lines. All QTLs identified in the SSR analysis were also identified in the combined marker analysis, although the former analysis always had much higher LOD scores (Table 3).

**Table 3:** Position and effects of quantitative trait loci (QTL) for seedling resistance to powdery mildew (PM) and adult plant resistance (APR) to PM and yellow rust (YR) in different environments (years).

Chromo-some	QTL	Isolate or year	Peak marker (cM)	Left marker (cM)	Right marker (cM)	LOD^a^^†^	Additive effect^b^^†^	R^2^^c^^†^
**PM resistance in seedling stage**								
**2A**	*QPm*.*haas-2A*	*Bgt*-6-11	*wsnp_JD_c289_450995*(164.2)	*BS00065434_51*(163.9)	*RAC875_c5082_841*(166.6)	7.91	0.49	6.6
**6A**	*QPm*.*haas-6A*	*Bgt*-6-11	*RAC875_c68978_220* (47.1)	*TA005690-1190* (45.4)	*RAC875_c48891_87*(51.2)	27.78 (85.11)	-1.48 (-0.95)	58.1 (80.6)
**PM resistance in adult stage**								
**2A**	*QPm*.*haas-2A*	2011	*wsnp_JD_c289_450995* (164.2)	*BS00065434_51*(163.9)	*BS00019095_51*(168.8)	6.66	5.86	18.6
	*QPm*.*haas-2A*	2013	*wsnp_JD_c289_450995* (164.2)	*BS00065434_51*(163.9)	*RAC875_c5082_841*(166.6)	2.99*	6.98	7.9
**3A**	*QPm*.*haas-3A*	2011	*BS00088103_51*(3.3)	*Xwmc11* (0.0)	*BS00088103_51* (3.3)	3.56	-4.00	9.3
**5A**	*QPm*.*haas-5A*	2012	*CAP8_c317_307*(229.3)	*RAC875_c9617_395*(225.1)	*wsnp_Ex_c54211_57168122*(237.6)	5.76	8.42	7.1
**6A**	*QPm*.*haas-6A*	2011	*RAC875_c68978_220* (47.1)	*TA005690-1190* (45.4)	*RAC875_c48891_87*(51.2)	4.10 (16.2)	-4.40 (-6.2)	10.7 (16.6)
	*QPm*.*haas-6A*	2012	*RAC875_c68978_220* (47.1)	*RAC875_rep_c69836_475*(43.2)	*RAC875_c48891_87*(51.2)	26.32 (108.1)	-25.59 (-30.0)	64.3 (77.2)
	*QPm*.*haas-6A*	2013	*RAC875_c68978_220* (47.1)	*RAC875_rep_c69836_475* (43.2)	*RAC875_c48891_87*(51.2)	5.92 (24.2)	-11.04 (-10.6)	17.0 (23.3)
**YR resistance in adult stage**								
**1B**	*QYr*.*haas-1B-1*	2010	*wsnp_Ex_c14_27570*(55.3)	*Tdurum_contig55639_241*(44.1)	*TA004407-0898* (65.7)	10.61 (24.99)	-13.60 (-12.2)	33.8 (23.3)
	*QYr*.*haas-1B-1*	2013	*wsnp_Ex_c14_27570*(55.3)	*Ex_c2725_1442* (49.6)	*BobWhite_c43322_203*(57.8)	6.43 (3.99)	-25.32 (-5.19)	27.7 (4.8)
**1B**	*QYr*.*haas-1B-2*	2013	*Excalibur_c43567_282*(34.4)	*Tdurum_contig50555_1144*(26.8)	*Excalibur_c54420_218* (35.1)	5.08	7.31	21.5
**2A**	*QYr*.*haas-2A*	2010	*Excalibur_c11491_1147*(9.8)	*Excalibur_c11491_1147*(9.8)	*BobWhite_c48481_81* (22.3)	4.34 (16.95)	7.73 (9.81)	11.5 (14.9)

a, QTLsignificant at P = 0.05 and * at P = 0.1 (suggestive).

b, negative value indicate resistance is derived from XK0106, positive from E07901.

c, indicate the additive variance explained by QTL.

†, numbers in brackets represent corresponding values of composite interval mapping on 388 DH lines with the SSR only map.

**Fig 2:**
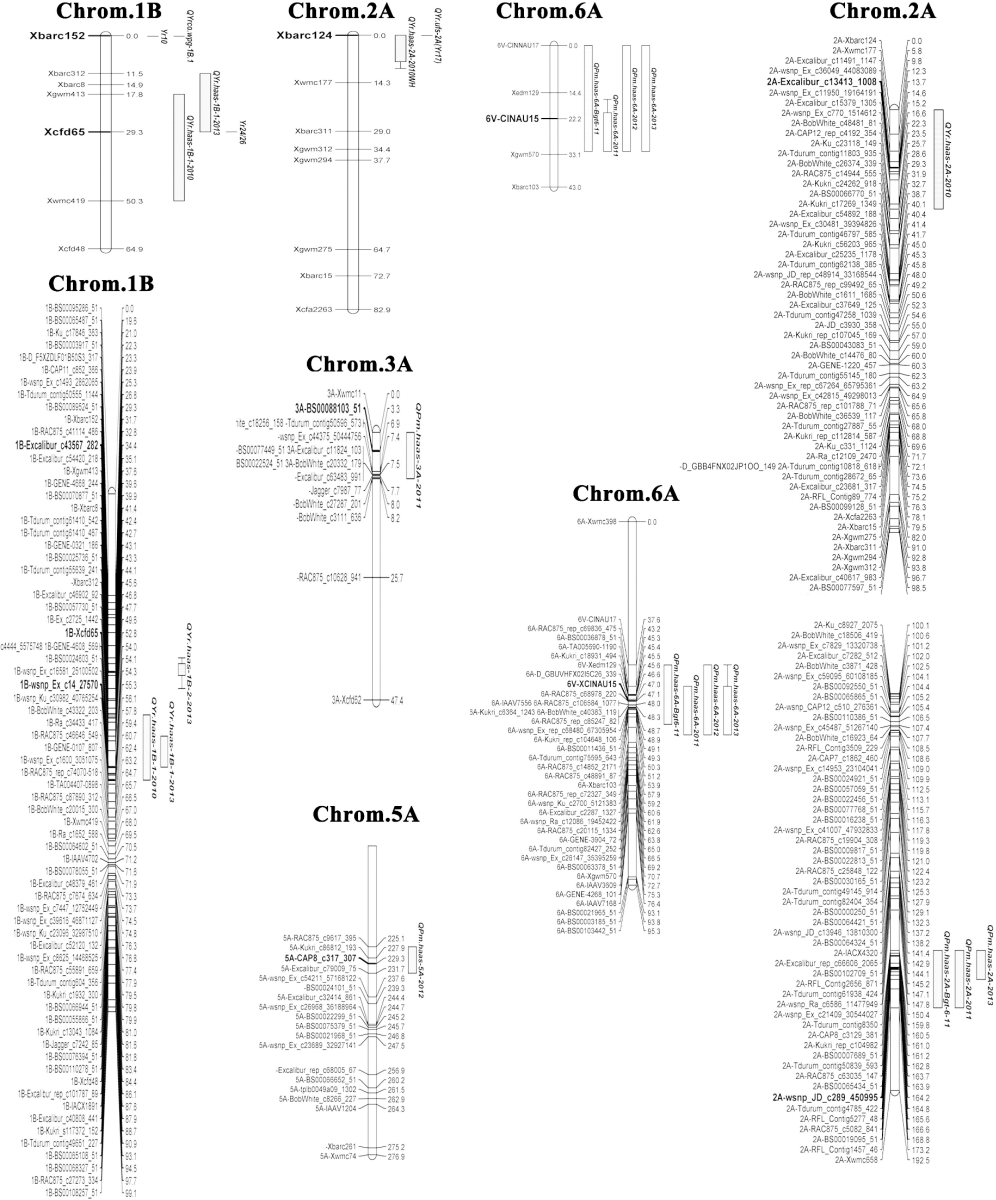
Linkage groups of wheat chromosomes showing SSR and SNP markers in QTL regions linked to resistance against powdery mildew (PM) and yellow rust (YR) in the DH population of E07901 × XK0106. Boxed QTLs show regions of significance at P≤0.05 and error bars at P≤0.1. Markers in bold highlight loci the peak marker for the associated QTL. Some related genes/QTLs were also shown in the LG by linked marker position in the consensus maps.

The most significant QTL detected for PMS was derived from XK0106 and was located on chromosome 6A. It was designated *QPm*.*haas-6A* and was identified in the seedling test (LOD 27.8) and in the field in 2011, 2012 and 2013 (respective LODs of 4.1, 26.3 and 5.9)(Table 3, Fig 2). *QPm*.*haas-2A* was derived from E07901 and was located on chromosome 2A. It was significant in the seedling test (LOD 7.9) and in the field in 2011 (LOD 6.7), and was a suggestive QTL (LOD 3.0) in 2013. *QPm*.*haas-3A* and *QPm*.*haas-5A* were significant in the field assays in 2011 and 2012 respectively. The former QTL, located on chromosome 3A, was derived from E07901 while the latter was on 5A and derived from XK0106. *QPm*.*haas-2A*, *QPm*.*haas-3A* and *QPm*.*haas-5* were not detected in the SSR map.

Three QTLs were detected in the YRS analysis, namely *QYr*.*haas-1B-1*, *QYr*.*haas-1B-2* and *QYr*.*haas-2A*. The first QTL was derived from XK0106 whilst the other two were from E07901. *QYr*.*haas-1B-1* was significant in both 2010 (LOD 10.6) and 2013 (LOD 6.4) while *QYr*.*haas-1B-2* was only significant in 2013 (LOD 5.1) and *QYr*.*haas-2A* only in 2010 (LOD 4.3). The QTLs for *QYr*.*haas-1B-1* and *QYr*.*haas-2A* were detected in both the SSR and the combined analyses (Table 3, Fig 2).

### Resistance evaluation of QTLs to PM and YR

The effects of the different PM QTLs were further tested in four environments in 2014 by selecting three DH lines each that contained various combinations of the different QTLs (Table 4). The four sites had sound epidemics although Wuhan and Xiangyang had heavier disease development. In lines that had only *QPm*.*haas-2A* or *QPm*.*haas-3A*, PMS was significantly reduced in Huanggang and Jingzhou, but not at the more heavily infected sites. Lines with only *QPm*.*haas-5A* had no effect against PMS in any environment. In nearly all environments, the combination of *QPm*.*haas-2A* with either *QPm*.*haas3A* or *QPm*.*haas-5A* significantly reduced disease severity below that of lines only containing *QPm*.*haas-2A*. Lines containing the combination of *QPm*.*haas-3A* and *QPm*.*haas-5A* fared no better than lines with *QPm*.*haas-3A* alone. The combination of all three QTLs reduced PMS when compared to lines containing the *QPm*.*haas*.*2A/QPm*.*haas-3A* combination, but were no better than the *QPm*.*haas*.*2A/QPm*.*haas-5A* combination.

**Table 4:** Mean severity of lines containing combinations of the indicated QTLs from the E07901 × XK0106 DH population, highlighting the additive effects of the QTLs to powdery mildew at multiple locations in 2014.

QTL / QTL combination	DH (No.)	Mean severity (%)^a^			
		Huanggang	Wuhan	Jingzhou	Xiangyang
**Null**	3	37.6 A*	73.6 A	46.5 A	66.1 A
***QPm*.*haas-2A***	3	14.4 B	55.3 AB	19.4 C	56.6 A
***QPm*.*haas-3A***	3	17.7 B	69.0 A	30.0 B	65.1 A
***QPm*.*haas-5A***	3	31.1 A	44.7 B	42.1 A	53.8 A
***QPm*.*haas-2A*/*QPm*.*haas-3A***	3	0.4 C	52.6 AB	11.0 C	37.9 B
***QPm*.*haas-2A*/*QPm*.*haas-5A***	3	0.3 C	25.7 C	10.0 C	18.9 C
***QPm*.*haas-3A*/*QPm*.*haas-5A***	3	14.2 B	55.0 AB	30.6 B	65.6 A
***QPm*.*haas-2A* /*QPm*.*haas-3A* /*QPm*.*haas-5A***	3	1.4 C	19.3 C	2.8 D	25.3 C

* Different letters following the mean indicates significant differences based on t-tests (*P* = 0.01).

**a,** Disease severity for powdery mildew at Meijiadun farm in Huanggang, Nanhu farm in Wuhan, Jiangbei farm in Jingzhou and Wolong farm in Xiangyang, Hubei Province.

A similar process was completed for YR where three DH lines were selected for each of the QTL combinations and tested for YRS in four environments in 2015. There were no lines that contained the combination of all three QTLs. Lines containing *QYr*.*haas-1B-1* showed immune responses in three environments but had the same score as the null lines in Gangu. *QYr*.*haas-1B-2* had a partial effect in reducing YRS, but this was again ineffective in Gangu while *QYr*.*haas-2A* had a partial effect that was significant in all environments. The combination of *QYr*.*haas-1B-1* with either QTL was not significantly different from the scores of *QYr*.*haas-1B-1* in Wuhan, Xiangyang and Kunming. However, *QYr*.*haas-1B-1* and *QYr*.*haas-2A* did reduce disease severity by more than *QYr*.*haas-2A* alone in Gangu. Finally, *QYr*.*haas-1B-2* and *QYr*.*haas-2A* acted additively to reduce disease severity in all environments by more than that observed when these QTLs were present alone (Table 5).

**Table 5:** Mean severity of lines containing combinations of the indicated QTLs from the E07901 × XK0106 DH population, highlighting the additive effects of the QTLs to yellow rust in multiple locations in 2015.

QTL / QTL combination	DH (No.)	Mean severity (%)^a^			
		Wuhan	Xiangyang	Kuming	Gangu
**Null**	3	53.0 A*	30.2 A	40.8 A	58.4 A
***QYr*.*haas-1B-1***	3	0.0 C	0.2 C	0.0 C	64.2 A
***QYr*.*haas-1B-2***	3	13.1 B	17.7 B	23.3 B	60.7 A
***QYr*.*haas-2A***	3	10.3 B	3.4 C	18.5 B	40.4 B
***QYr*.*haas-1B-1*/*QYr*.*haas-1B-2***	3	1.9 C	0.1 C	0.0 C	50.3 AB
***QYr*.*haas-1B-1*/*QYr*.*haas-2A***	3	3.1 C	0.0 C	0.3 C	21.8 C
***QYr*.*haas-1B-2*/*QYr*.*haas-2A***	3	1.7 C	0.5 C	1.7 C	24.0 C

* Different letters following the mean indicates significant differences based on t-tests (*P* = 0.01).

**a,** Disease severity for yellow rust at Nanhu farm in Wuhan and Wolong farm in Xiangyang, Hubei Province, Taoyuan farm in Kunming, Yunnan Province and Gangu farm in Gangu, Gansu Province.

## Discussion

This study identified resistance loci to PM and YR in a DH population derived from a cross between E07901 and XK0106 and evaluated the effectiveness of these loci in different combinations and environments. One QTL (*QPm*.*haas-6A*) for PMS was detected at seedling and adult plant stages in all environments and its seedling reactions and genomic location identified it as *Pm21*. A further three QTLs were found for PMS, *QPm*.*haas-2A*, *QPm*.*haas-3A* and *QPm*.*haas-5A*. *QPm*.*haas-2A* was significant in the seedling test and two field environments and is likely a previously unidentified gene. The YRS study identified three QTLs with *QYr*.*haas-1B-1* having a strong effect in both QTL field environments, while *QYr*.*haas-1B-2* and *QYr*.*haas-2A* were only effective in 2013 and 2010 respectively. Seedling pathotype testing and the genomic location also suggest *QYr*.*haas-1B-2* had not previously been identified, although *QYr*.*haas-1B-1* is likely *Yr24/26* and *QYr*.*haas-2A* is *Yr17*. A novel strategy was developed to understand how the different QTLs interacted in different environments. Specific lines from the DH population that contained various combinations of QTLs were used as an effective means by which to characterize genotype × environment interactions of these loci in multiple environments.

### Comparison of QTLs to known resistance genes

The major seedling resistance of *Pm21* was located on chromosome 6A and associated with well characterized markers, in particular, *6V-CINAU15*, which is deemed a functional marker for this gene [31]. Indeed this marker, along with the other *Pm21* associated markers of *6V-CINAU17* and *Xedm129* [32,33], all occurred within the QTL region of *QPm*.*haas-6A*. Furthermore, SNP markers also associated with this QTL, *RAC875_rep_c69836_475*, *RAC875_c48891_87* and the peak marker *RAC875_c68978_220*, have also been placed in this region through consensus maps [34]. This designation is also supported by seedling tests to 16 *B*. *graminis* isolates where XK0106 was immune to all isolates and matched the pattern produced by Yangmai5/sub.6v, a line with the *Pm21* containing translocation. *Pm21* was introduced into common wheat through the translocation T6VS-6AL derived from 6VS of *Haynaldia villosa* (2n = 2x = 14, VV) [38]. As it gives high levels of resistance to PM, the T6VS 6AL translocation has been widely used in breeding programs since 2002, particularly in powdery mildew prevalent provinces including Sichuan, Guizhou, Gansu and Jiangsu. Cultivars released such as Neimai8, Neimai836, Neimai10, Neimai11 and Mianmai39 have all been widely planted in Sichuan Province and contain *Pm21* [31]. XK0106 originated from Sichuan Province and as its resistance has now been confirmed to contain *Pm21*, it must also be derived from a T6VS-6AL translocation line.

There have been two other introgressions containing PM resistance genes on chromosome 6A, *MIRE*, introgressed from *T*. *dicoccum* [39] and *MIG* from *T*. *dicoccoides* [40]. Map positions clearly differentiate these loci from *Pm21* [40]. These 6A introgressions show the value of wild species in contributing useful resistances to the common wheat gene pool.

*QPm*.*haas-2A* was flanked by the markers *BS00065434_51* and *RAC875_c5082_841* with the peak marker being *wsnp_JD_c289_450995* (position 164.2 cM Fig 2A). A consensus map of Wang et al.[34] placed this QTL towards the telomere of 2AL. Several QTLs including *QPm*.*inra-2A* [41], *QPm*.*vt-2A* [42,43], *Qpm*.*ttu-2A* [43] and *Qpm*.*crag-2A* [44] have been identified on chromosome 2A. Li et al. [18] reviewed all PM QTLs and located *QPm*.*inra-2A* on the short arm of 2A. *QPm*.*vt-2A* was near the centromere on 2AL and was associated with *Xgwm312* [42]. On our map this marker is 90 cM (Position 93.8 cM Fig 2A) from the QTL peak marker. The map positions therefore clearly differentiate *QPm*.*haas-2A* from the two aforementioned QTLs. *Qpm*.*ttu-2A* and *Qpm*.*crag-2A* were located near the telomere of 2AL with the former being tightly linked to *Xwmc658* [43]. Again in our map this marker is over 28 cM from the QTL peak (Position 192.5 cM Fig 2A). Mingeot et al. [44] mapped *Qpm*.*crag-2A* to the same locus as the seedling resistance gene *Pm4b*, and described the QTL as a residual effect of the defeated gene. S1 Table shows numerous differences between the seedling reactions of Armada, a *Pm4b* carrying line, and E07901, the *QPm*.*haas-2A* donor. This also indicates that *Qpm*.*haas-2A* is different from *Qpm*.*crag-2A* (*Pm4b*) and is therefore likely a new QTL for PM.

The *Qpm*.*haas-3A* and *Qpm*.*haas-5A* loci had minor effects in 2011 and 2012, respectively. It was difficult to judge the relationship of these QTL with other known QTLs on chromosome 3A and 5A as there was an absence of shared markers between our maps and other reported maps. However these QTLs and their associated markers could be useful to pyramid minor genes for durable resistance.

*QYr*.*haas-1B-1* on chromosome 1B was detected in both 2010 and 2013 and is likely to be *Yr24/26* due to its map location and virulence testing. *Yr24/26* has been mapped to chromosome 1BL with associated markers *Xwe173* and *Xbarc181* [23,45,46,47]. The Somers consensus SSR map ([30];http://wheat.pw.usda.gov/GG3) places the peak SSR marker of *QYr*.*haas-1B-1*, *Xcfd65*, at the same position as *Xgwm11* and *Xgwm18*. Furthermore, the resistance patterns of XK0106 (*QYr*.*haas-1B-1* donor) to five Chinese *Pst* races in seedling tests were similar to that of the Avocet NIL line containing *Yr24/26* (S2 Table). These data provide strong evidence of the *Yr24/26* supposition.

*Excalibur_c43567_282* and *Xgwm413* were associated with *QYr*.*haas-1B-2* and both have been located to chromosome 1BS on consensus genetic maps [34]. Apart from *Yr24/26*, there are several other genes that have been identified on this chromosome including *Yr10*[43], *Yr15*[44], *YrCH42*, *YrH52* [48], *Yr29/Lr46*[49], *Yr64*and*Yr65*[9]. More recently, *Yr24/26* and *YrCH42* have been shown to be identical due to their similar genomic position and reaction patterns against 26 *Pst* isolates [45]. The closest known YR genes to *QYr*.*haas-2B-2* are *Yr10* [48] and *Yr15* [49] and both of these were clearly differentiated from *QYr*.*haas-1B-2* through seedling pathotype tests. Despite detailed mapping, *YrH52* could not be separated from *Yr15* and as both are derived from *T*. *dicoccoides*, they have yet to be clearly identified as different loci [50,51]. E07901 (*QYr*.*haas-1B-2* donor) was MS to S against five pathogens tested, while the *Yr10* NIL had a resistant reaction to CYR29 and Sul1-4 and the *Yr15* NIL had a resistant reaction to all pathotypes. Furthermore, these genes are rarely used in current breeding programs in China [15,25] with *Yr15* being derived from *T*. *dicoccoides* [49,50] and presents with significant linkage drag. *QYrco*.*wpg-1B*.*1* has also been reported in this region as a QTL that had both race specific seedling reactions and robust APR [52]. The marker *Xpsp3000* mapped 2–4.4 cM proximal to the seedling reaction QTL *QYrco*.*wpg-1B*.*1* and 1.2cM from *Yr10* [53], suggesting a very similar location for these loci, although pedigree data suggested that they were different genes. *QYr*.*haas-1B-2* could be differentiated from *QYrco*.*wpg-1B*.*1* with the marker *Xgwm413*. This marker was 3.2 cM proximal to *QYr*.*haas-1B-2*, yet 44 cM proximal to *QYrco*.*wpg-1B*.*1* [52]. Furthermore, *Xpsp3000* and *Xgwm413* are 59 cM apart on the Somers Consensus map [30].

*QYr*.*haas-1B-2* is unlikely to be any of the other gene identified on 1B. *YrH52* is from *T*. *dicoccoides* and has only been introgressed into *T*. *durum* with the gene containing segment suffering from negative crossover interference [50]. *Yr29/Lr46* is a single locus that is located towards the telomere of chromosome 1BL [54] while *Yr64* and *Yr65* have only recently been introduced from *T*. *durum* and are yet to be deployed in hexaploid wheat [9]. All of these data suggest that *QYr*.*haas-1B-2* is likely a new QTL for YR.

Another QTL for YR was mapped to the telomeric end of chromosome 2AS (*QYr*.*haas-2A*) with the SSR markers *Xbarc124* and *Xwmc177*. Several YR resistance genes or QTLs have been reported on chromosome 2AS including the race-specific gene *Yr17* [41], as well as *QYr*.*ufs-2A* [55], *QYr*.*uga-2AS* [56] and *QYr*.*ucw-2AS* [57]. *Yr17* was also located towards the teleomere of 2AS, and was associated with *Xgwm636* [41]. This marker, along with the two *QYr*.*haas-2A* associated markers, are within 7 cM of each other on the Somers consensus map [30]. As *QYr*.*haas-2A* could not be differentiated from *Yr17* by neither map position nor seedling assays, we made the conservative assumption that *QYr*.*haas-2A* was likely to be *Yr17*, although testing with an avirulent pathotype would be required to confirm this.

### Resistance evaluation of QTLs to PM and YR

Durable resistance to rust diseases under severe epidemics has been achieved through the combining of three minor loci to control leaf rust [58] and up to five to control yellow rust [59]. Although *QPm*.*haas-6A* (*Pm21*) is still an effective major gene, given its wide-spread deployment over a large area in numerous Chinese cultivars, there is a strong possibility that it will breakdown in the coming years. This study investigates the role the minor QTLs could play in the absence of *Pm21*. The additive effect of various combinations of these minor QTLs were investigated in several environments by selecting three lines that contained each of the various QTL combinations. This novel approach allowed detailed investigations of the major QTLs without having to grow out the entire mapping population. This has the advantage of being able to grow more replicates of each line, increasing the plot size and being able to take more detailed notes on each plot. These factors result in more accurate scores for each line tested. There is a disadvantage in not being able to identify new QTLs that may be environment specific, however such QTLs are often relatively minor in effect.

Disease pressure had a significant impact upon whether single loci could reduce disease severity. Huanggang and Jingzhou had the lowest disease severity of powdery mildew as evidenced by scores of lines with the null QTL combination. In these low severity sites, both *QPm*.*haas-2A* and *QPm*.*haas-3A* significantly reduced disease severity, yet had no effect at the high disease sites of Wuhan and Xiangyang. *QPm*.*haas-5A* had little effect when present by itself (Table 4). It is doubtful however that any of these loci in isolation would provide much protection of yield even under moderate disease pressures.

Combinations of QTLs identified interesting additive effects. *QPm*.*haas-2A* and *QPm*.*haas-5A* combined well and reduced disease severity in all environments despite the lack of effect of *QPm*.*haas-5A* alone. This is indicative of an epistatic interaction between these two loci. Contrastingly, the *QPm*.*haas-3A/QPm*.*haas-5A* combination was no better than the 3A QTL alone. Such as situation has been previously observed in another YR QTL study where both QTLs on chromosomes 3D and 5D gave moderate protection in isolation and that was no different than the protection provided when they were combined. However these QTLs were clearly additive with all other loci [17]. Such an interaction suggests they may share part of a similar pathway in their respective defense responses and the two PM QTLs identified herein follow a similar pattern.

Lines with all three PM QTLs fared little better than lines with the *QPm*.*haas-2A/QPm*.*haas-5A* combination, again highlighting the non-additive effect of *QPm*.*haas-3A*. This is important information for breeders who wish to pursue additive resistances to achieve durability. The two minor genes with additive effects that have been combined in this study reduced disease severity by two thirds in the stronger epidemic environments and further reduced it to negligible levels in less severe sites. It is hoped that, as is the case for the rust diseases, by recombining two to three more loci, near-immunity may be reached. Indeed such loci are readily available. A number of pleiotropic, durable APR loci that give intermediate levels of resistance to leaf rust, yellow rust and stem rust have been identified, and recent work has shown they also have effects against PM. These loci have been termed *Lr34/Yr18/Sr57/Pm38* [60], *Lr46/Yr29/Sr58/Pm39* [61] and *Lr67/Yr46/Sr55/Pm46* [62]. Sound molecular markers are available and it would be a straight-forward breeding exercise to recombine these with the QTLs described herein, in an attempt to generate near-immune lines.

A similar approach of selecting lines with various QTLs was adopted with YR. This gave insights not only into additive effects of different loci, but also of pathogen composition in the various environments. This was most clearly demonstrated with the prevalence of *Pst-V26* in Gangu where *QYr*.*haas-1B-1* containing lines scored high, but were immune at other sites. Virulence to *Yr24/26* was first detected in China on wheat cultivar Chuanmai42 in the Sichuan Basin in 2008 [63]. Virulence was subsequently shown in Gansu on lines 92R137 and Guinong22 [64], and steadily increased throughout the region [65]. This race is not yet dominant in populations in Wuhan, Xiangyang and Kunming, and *QYr*.*haas-1B-1* (*Yr24/26*) showed excellent resistance in these three sites in 2015. Furthermore, the very low scores of lines combining *QYr*.*haas-1B-1* with other QTLs reflected the overriding response strong seedling resistances have when combined with intermediate levels of resistance. It seems likely that *Pst-V26* also has virulence to *QYr*.*haas-1B-2* as this QTL was not only ineffective in isolation in Gangu, but so were the lines that combined it with the *Yr24/26* locus.

*QYr*.*haas-1B-2* and *QYr*.*haas-2A* had significant effects in reducing YR severity but did not create the immune response as observed with *Yr24/26*. The *QYr*.*haas-2A* effect is consistent with *Yr17* where resistance is often incomplete, which can be influenced by genetic background and growing conditions [7]. This locus was still effective in Gangu and partially reduced disease severity. Furthermore, when *Yr17* was combined with either *Yr24/26* or *QYr*.*haas-1B-2*, disease scores in Gangu were further lowered. This suggests that there were mixed isolates in the field, some with virulence to *Yr17* and other with virulence to the other QTLs.

### Genetic map and the number of QTL detected

In this study, two marker sets were used to empirically investigate the effectiveness of population size and marker density in identifying QTLs. A QTL analysis was initially undertaken with a sparse genetic map (117 SSR markers) but with a large population size (388 lines). A subsequent analysis used a high density genetic map (11,330 markers) with a small population size (80 lines). The QTLs detected in the SSR map spanned much longer chromosome segments and this is not surprising given the low marker density of 10.6 cM per marker compared to the much higher density in the SNP map (2.5 cM per marker). Furthermore, the LOD scores in the SSR map were generally two to four times higher than in the SNP map and is a reflection of the vastly larger population sizes giving much greater confidence in the QTLs observed. However, most telling was the total number of QTLs identified, with seven different loci proving significant in the SNP map, while only three were apparent in the SSR map. This is again due to the greater genome coverage afforded by the SNP map as additionally identified QTLs were mostly in regions without any SSR coverage. The only exception was *QYr*.*haas-1B-2* that was derived from E07901. However this was close to the XK0106 derived *QYr*.*haas-1B-1* which provided immunity that would mask the more minor effects of the former QTL in the sparse SSR map.

In conclusion, major seedling resistance genes were found for both pathogens and these corresponded to *Pm21*, *Yr24/26* and *Yr17*. Two new QTLs were likely identified in *QPm*.*haas-2A* and *QYr*.*haas-1B-2*, along with two other minor PM QTLs. QTL combination studies showed the ability to pyramid some of the PM QTLs is a starting point for developing near-immune lines based on QTLs, and gave insights into the pathogen populations in the YR sites. Finally, empirical testing of overlapping mapping populations with different marker densities and population sizes highlighted the usefulness of SNP platforms, even in relatively small populations.

## Supporting information

S1 Table Reaction type of sixteen differentials *Bgt* isolates on a set of known *Pm* genes lines and on the bi-parent. (DOC)

S2 Table Reaction types pattern of 5 Chinese *Pst* toXK0106, E07901 and 4*Yr* single-gene lines containing known gene in chromosome 1B and 2A. (DOC)

S3 Table Molecular markers linkage groups and map distances of the E07901 × XK0106 wheat map. (XLSX)

S4 Table Original dataset of molecular marker linkage groups of the E07901 × XK0106 wheat mapping population. (XLSX)

## Abbreviations

APR: Adult Plant Resistance
ANOVA: Analysis of Variance
*Bgt*: *Blumeria graminis* f.sp. *tritici*
DH: Doubled Haploid
LG: Linkage Group
LOD: Log-likelihood
IT: InfectionType
PM: Powdery Mildew
PMS: Powdery Mildew Severity
*Pst*: *Puccinia striiformis* f.sp. *tritici*
QTL: Quantitative Trait Loci
YR: Yellow Rust
YRS: Yellow Rust Severity
