# Supplementary material for: Identification and evaluation of resistance to powdery mildew and yellow rust in a wheat mapping population
Source: PLoS One. 2017 May 23;12(5):e0177905. doi: 10.1371/journal.pone.0177905 (PMC5441593; doi:10.1371/journal.pone.0177905)
Supplement: S1 Table — (DOC) [file pone.0177905.s001.doc]

| No. | line | *Pm* | *Blumeria graminis tritici* isolates, *Bgt* | | | | | | | | | | | | | | | |
| --- | --- | --- | --- | --- | --- | --- | --- | --- | --- | --- | --- | --- | --- | --- | --- | --- | --- | --- |
|  |  |  | E01 | E02 | E03 | E05 | E06 | E07 | E09 | E11 | E13 | E15 | E16 | E17 | E18 | E20 | E21 | E26 |
| 1 | chancellor | — | 4 | 4 | 4 | 4 | 4 | 4 | 4 | 4 | 4 | 4 | 4 | 4 | 4 | 4 | 4 | 4 |
| 2 | Axminster/8cc | *Pm1* | 4 | 1 | 4 | 4 | 0 | 0 | 4 | 1 | 1 | 4 | 4 | 4 | 4 | 4 | 4 | 4 |
| 3 | Ulka/8cc | *Pm2* | 1 | 0 | 0 | 0 | 0 | 0 | 1 | 1 | 0 | 1 | 0 | 0 | 4 | 4 | 4 | 0 |
| 4 | Assosan/8cc | *Pm3a* | 4 | 1 | 0 | 4 | 0 | 4 | 4 | 3 | 1 | 3 | 4 | 0 | 4 | 4 | 3 | 4 |
| 5 | Chul/8cc | *Pm3b* | 4 | 0 | 4 | 4 | 0 | 0 | 4 | 1 | 1 | 0 | 4 | 4 | 4 | 4 | 4 | 0 |
| 6 | Sonoara/8cc | *Pm3c* | 4 | 3 | 4 | 4 | 4 | 4 | 4 | 4 | 3 | 0 | 4 | 4 | 4 | 4 | 4 | 2 |
| 7 | Kolibri | *Pm3d* | 1 | 0 | 0 | 0 | 0 | 0 | 0 | 2 | 0 | 0 | 0 | 0 | 4 | 4 | 4 | 0 |
| 8 | W150 | *Pm3e* | 3 | 3 | 0 | 3 | 4 | 4 | 2 | 4 | 4 | 3 | 4 | 4 | 3 | 2 | 3 | 4 |
| 9 | Michigen | *Pm3f* | 4 | 0 | 0 | 4 | 4 | 0 | 0 | 4 | 3 | 3 | 0 | 0 | 3 | 4 | 4 | 4 |
| 10 | Khapli/8cc | *Pm4a* | 0; | 0 | 0 | 0 | 0 | 0 | 0 | 0 | 0 | 4 | 0 | 0 | 3 | 4 | 4 | 0 |
| 11 | Armada | *Pm4b* | 0; | 0; | 0 | 0 | 4 | 1 | 0 | 0 | 0 | 0 | 0 | 0 | 4 | 4 | 4 | 0 |
| 12 | Hope/8cc | *Pm5* | 4 | 3 | 4 | 4 | 4 | 4 | 3 | 4 | 4 | 4 | 3 | 4 | 3 | 3 | 4 | 4 |
| 13 | Aquila | *Pm5(Mli)* | 0 | 0 | 0 | 4 | 0 | 0 | 0 | 0 | 0 | 0 | 0 | 3 | 3 | 3 | 4 | 4 |
| 14 | Timgalen | *Pm6* | 2 | 3 | 4 | 4 | 3 | 4 | 3 | 3 | 1 | 2 | 2 | 4 | 3 | 1 | 4 | 3 |
| 15 | CII4189 | *Pm7* | 4 | 4 | 4 | 4 | 4 | 4 | 4 | 4 | 4 | 4 | 4 | 4 | 3 | 4 | 4 | 4 |
| 16 | Kavkaz | *Pm8* | 3 | 4 | 4 | 4 | 4 | 4 | 4 | 4 | 4 | 3 | 4 | 4 | 3 | 4 | 4 | 4 |
| 17 | R4A | *Pm13* | 1 | 1 | 1 | 1 | 3 | 1 | 1 | 1 | 1 | 1 | 2 | 1 | 0 | 2 | 3 | 1 |
| 18 | Amigo | *Pm17* | 3 | 0 | 4 | 4 | 4 | 3 | 3 | 4 | 3 | 1 | 4 | 3 | 3 | 3 | 4 | 4 |
| 19 | Bounty | *Pm19* | 3 | 4 | 3 | 3 | 4 | 4 | 3 | 4 | 4 | 1 | 4 | 3 | 3 | 4 | 3 | 4 |
| 20 | PI583795 | *Pm20* | 4 | 4 | 0 | 1 | 0 | 4 | 4 | 4 | 0 | 0; | 4 | 4 | 0 | 1 | 1 | 0 |
| 21 | Yangmai5/sub.6v | *Pm21* | 0 | 0 | 0 | 0 | 0 | 0 | 0 | 0 | 0 | 0 | 0 | 0 | 0 | 0 | 0 | 0 |
| 22 | Nc96BGTA3 | *Pm22* | 1 | 0 | 0 | 1 | 0 | 4 | 0 | 1 | 1 | 0 | 1 | 4 | 1 | 1 | 1 | 0 |
| 23 | Xiaobaidongmai | *XBD* | 0 | 2 | 0 | 1 | 1 | 3 | 4 | 4 | 4 | 0 | 0 | 1 | 0 | 0 | 3 | 0 |
| 24 | Maris Dove | *Pm2+Mld* | 1 | 0; | 0 | 0 | 0 | 0 | 1 | 0 | 0; | 0 | 0 | 0 | 0 | 4 | 4 | 0 |
| 25 | Brock | *Pm2+Ta* | 4 | 4 | 0 | 4 | 4 | 4 | 0 | 4 | 4 | 0 | 0 | 4 | 0 | 3 | 4 | 4 |
| 26 | CII2632 | *Pm2+6* | 2 | 0 | 0 | 0 | 0 | 0 | 0 | 1 | 0 | 0 | 0 | 0 | 3 | 3 | 3 | 0 |
| 27 | Maris Huntsman | *Pm2+6* | 0 | 0 | 0 | 0 | 0 | 0 | 0 | 0 | 0 | 0 | 0 | 0 | 3 | 4 | 4 | 0 |
| 28 | Baimain3 | *Pm4+8* | 3 | 1 | 0 | 0 | 0 | 0 | 0 | 0 | 1 | 0 | 0 | 0 | 3 | 4 | 4 | 3 |
| 29 | Misson | *Pm4b+Mli* | 0 | 0 | 1 | 0 | 4 | 1 | 0 | 4 | 4 | 0 | 0 | 1 | 1 | 4 | 4 | 4 |
| 30 | Coker983 | *Pm5+6* | 0 | 0 | 0 | 3 | 0 | 1 | 0 | 0 | 0 | 0 | 0 | 4 | 0 | 2 | 3 | 0 |
| 31 | Normandie | *Pm1+2+9* | 1 | 0 | 0 | 3 | 3 | 4 | 0 | 3 | 4 | 0 | 4 | 3 | 1 | 4 | 3 | 4 |
| 32 | XK0106 |  | 0 | 0 | 0 | 0 | 0 | 0 | 0 | 0 | 0 | 0 | 0 | 0 | 0 | 0 | 0 | 0 |
| 33 | E07901 |  | 1 | 4 | 4 | 4 | 4 | 4 | 3 | 4 | 4 | 4 | 4 | 3 | 4 | 4 | 4 | 3 |
