# Supplementary material for: Identification and evaluation of resistance to powdery mildew and yellow rust in a wheat mapping population
Source: PLoS One. 2017 May 23;12(5):e0177905. doi: 10.1371/journal.pone.0177905 (PMC5441593; doi:10.1371/journal.pone.0177905)
Supplement: S2 Table — (DOC) [file pone.0177905.s002.doc]

| Line | *Yr* gene | *Puccinia striiformis tritici* (*Pst*) race | | | | |
| --- | --- | --- | --- | --- | --- | --- |
|  |  | CYR29 | CYR32 | CYR33 | Su11-4 | V26 |
| AvSYr10NIL | *Yr10* | 0 | 2 | 2 | 0 | 8 |
| AvSYr15NIL | *Yr15* | 0 | 0 | 0 | 0 | 0 |
| AvSYr17NIL | *Yr17* | 7 | 9 | 8 | 5 | 8 |
| AvSYr26NIL | *Yr26* | 2 | 2 | 1 | 1 | 9 |
| XK0106 | unknown | 1 | 3 | 2 | 1 | 8 |
| E07901 | unknown | 4 | 5 | 6 | 4 | 9 |
| Mingxian169 | - | 9 | 9 | 9 | 9 | 9 |
